# Statistics of Mortality

**Published:** 1855-03

**Authors:** 


*Statistics of Mortality.*

The following tables, compiled from the records of the City Sexton, are quoted mainly from the columns of one of the daily papers of our city. The first shows the number of deaths in each month for a series of eight years, as follows, viz.:

| | '47. | '48. | '49. | '50. | '51. | '52. | '53. | '54. |
|---|---|---|---|---|---|---|---|---|
| January, | 33. | 26. | 52. | 60. | 30. | 48. | 58. | 112. |
| Feb., | 23. | 31. | 62. | 57. | 29. | 43. | 61. | 101. |
| March, | 32. | 41. | 36. | 53. | 35. | 42. | 81. | 96. |
| April, | 19. | 31. | 49. | 50. | 35. | 63. | 59. | 103. |
| May, | 26. | 48. | 127. | 43. | 45. | 70. | 73. | 147. |
| June, | 27. | 41. | 173. | 27. | 35. | 94. | 82. | 331. |
| July, | 53. | 41. | 411. | 240. | 67. | 179. | 111. | 933. |
| August, | 65. | 65. | 242. | 466. | 237. | 468. | 152. | 731. |
| Septemb'r, | 87. | 60. | 164. | 174. | 175. | 300. | 191. | 563. |
| October, | 55. | 63. | 97. | 70. | 48. | 194. | 114- | 408. |
| Novemb'r, | 50. | 65. | 61. | 46. | 45. | 86. | 90. | 200. |
| December, | 30. | 43. | 42. | 49. | 54 | 75. | 121. | 108. |
| | 500. | 655. | 1516. | 1335. | 836. | 1662. | 1193. | 3833. |

The larger proportional mortality, in the years 1849, 50, 52, and 54, was owing to the severe prevalence of epidemic cholera. The same disease prevailed, though to a very limited extent, in 1851, while 1853 was almost wholly exempt from its ravages.

The following table will show the mortality of four principal Eastern cities for two consecutive years, viz.:

| Deaths. | Philadelphia. | New York. | Boston. | Baltimore. |
|---|---|---|---|---|
| 1853 | 9,750 | 21,864 | 4,369 | 5,117 |
| 1854 | 11,811 | 28,458 | 4,418 | 5,738 |
| Increase in '54, | 2,061 | 6,594 | 49 | 621 |

From this it will be seen that the year 1854 has been one of unusual mortality in most of the eastern, as well as the western cities. The ratio of deaths to the population in Chicago for a series of eight years is as follows, viz:

| | | | | | | |
|---|---|---|---|---|---|---|
| 1847 | Population, | 16,859 | Deaths, | 500 | Ratio, | 1 in 32,4 |
| 1848 | " | 19,724 | " | 655 | " | 1 in 30,2 |
| 1849 | " | 23,047 | " | 1516 | " | 1 in 15,2 |
| 1850 | " | 28,620 | " | 1335 | " | 1 in 21,5 |
| 1851 | " | 32,000 | " | 836 | " | 1 in 38,3 |
| 1852 | " | 38,733 | " | 1662 | " | 1 in 24,1 |
| 1853 | " | 60,662 | " | 1193 | " | 1 in 50,3 |
| 1854 | " | 70,000 | " | 3833 | " | 1 in 18,3 |

These figures show an annual average ratio of mortality for the whole eight years, of about one in 29, which compared with several eastern cities would exhibit the following results, viz:

| | | | | | | | |
|---|---|---|---|---|---|---|---|
| Philadelphia, | 1 | death | in | every | 42 | inhabitants | annually. |
| Baltimore, | 1 | " | " | " | 37 | " | " |
| Boston, | 1 | " | " | " | 36 | " | " |
| Chicago, | 1 | " | " | " | 29 | " | " |
| New York, | 1 | " | " | " | 25 | " | " |

It will be seen by this table that the ratio of deaths in New York is much higher than the average in this city, and in both the ratio considerably exceeds that of Philadelphia, Baltimore, and Boston. This is owing entirely to the greater proportional number of *immigrants*, constantly arriving in the two former cities. In this city, for instance, full one half of the entire population are foreign born, and there is constantly present a large number of new comers, constituting a floating population of several thousand, many of whom are yet under the debilitating influences of a long voyage, and destitute of every comfort or convenience of life. It is from the ranks of these poor people that the cemeteries of New York and Chicago receive their large excess of victims. This truth will be further illustrated by comparing the ratio of deaths in the several cities from some of the more important diseases.—Thus the ratio of deaths in 1854 from

| | Philadelphia | Baltimore. | Boston. | Chicago. | N. York |
|---|---|---|---|---|---|
| Consumption. | 1 in 359 | 1 in 226 | 1 in 209 | 1 in 272 | 1 in 209 |
| Chol. Mor. & Diarr. | 1 in 500 | 1 in 242 | 1 in 993 | 1 in 262 | 1 in 219 |
| Dysentery, | 1 in 1014 | 1 in 830 | 1 in 855 | 1 in 300 | 1 in 755 |
| General Fevers, | 1 in 3012 | 1 in 1850 | 1 in 1568 | 1 in 500 | 1 in 1240 |
| Inflamation Lungs, | 1 in 1096 | 1 in 1391 | 1 in 356 | 1 in 1892 | 1 in 541 |
| Cholera, | 1 in 832 | 1 in —— | 1 in 628 | 1 in 47 | 1 in 254 |

By examining this table, the reader will see at a glance that the excess of mortality in Chicago and New York, is chiefly from affections of the bowels, included under the heads of cholera morbus, diarrhœa, dysentery, and epidemic cholera. In Chicago almost half of the entire mortality was from the latter disease alone; while the ratio of deaths from diseases of the respiratory organs was much below either of the other cities.

We would gladly include in these tables of comparative morality, the cities of Buffalo, Detroit, Milwaukie, Cincinnatti, St. Louis, and New Orleans, but the necessary figures are not at our command.

